# Ovarian cancer stem cells and targeted therapy

**DOI:** 10.1186/s13048-019-0588-z

**Published:** 2019-12-06

**Authors:** Vahideh Keyvani, Moein Farshchian, Seyed-Alireza Esmaeili, Hadi Yari, Meysam Moghbeli, Seyed-Reza Kazemi Nezhad, Mohammad Reza Abbaszadegan

**Affiliations:** 1 Medical Genetics Research Center, Mashhad University of Medical Sciences, Mashhad, Iran; 2 Department of Biology, Faculty of Science, Shahid Chamran University of Ahvaz, Ahvaz, Iran; 3 Stem Cell and Regenerative Medicine Research Group, Academic Center for Education, Culture and Research (ACECR), Khorasan Razavi Branch, Mashhad, Iran; 4 Immunology Research Center, Bu‐Ali Research Institute, Mashhad University of Medical Sciences, Mashhad, Iran; 5 Department of Immunology, Faculty of Medicine, Mashhad University of Medical Science, Mashhad, Iran; 6 Human Genetics Division, Medical Biotechnology Department, National Institute of Genetics Engineering and Biotechnology, Tehran, Iran

**Keywords:** Cancer stem cell, Ovarian cancer, Isolation, Detection, Drug resistance

## Abstract

**Background:**

Ovarian cancer has the highest ratio of mortality among gynecologic malignancies. Chemotherapy is one of the most common treatment options for ovarian cancer. However, tumor relapse in patients with advanced tumor stage is still a therapeutic challenge for its clinical management.

**Main body:**

Therefore, it is required to clarify the molecular biology and mechanisms which are involved in chemo resistance to improve the survival rate of ovarian cancer patients. Cancer stem cells (CSCs) are a sub population of tumor cells which are related to drug resistance and tumor relapse.

**Conclusion:**

In the present review, we summarized the recent findings about the role of CSCs in tumor relapse and drug resistance among ovarian cancer patients. Moreover, we focused on the targeted and combinational therapeutic methods against the ovarian CSCs.

## Background

Ovarian cancer is the seventh most common cancer and the fifth leading cause of cancer related deaths among women globally (15–20 per 100,000) [1]. Ovarian cancer is a heterogeneous malignancy with different clinical development. Such a large heterogeneity is the result of various biological processes that underlie different types of ovarian cancers. Contrary to the classic view that different ovarian cancer histotypes are caused by metaplastic changes of a single tissue, only a subset of epithelial ovarian cancers develops within the ovarian surface epithelium (OSE) [2]. Majority of tumors originate in non-ovarian areas [3]. Ovarian cancer has significant challenges due to its intrinsic molecular heterogeneity which is associated with different tumor histotypes [4]. Different types of ovarian tumors have different phenotypes, molecular biology, etiology, progression, and prognosis [5]. Ovarian cancer has two main histological sub types including surface epithelial stromal and sex cord stromal cells [6]. Surface epithelial cells (OSE) or intra epithelial carcinomas (STIC) are the most important origins of ovarian cancers. The epithelial type involves about 90% of ovarian tumors, and is categorized into genetically sustained with low grade serous and invasive genetically ephemeral with high grade serous [7]. The highest prevalence of ovarian cancer is observed in eastern Asian countries and central America [8]. Lifestyle changes have decreased the rate of mortality in western countries [9]. Epidemiological studies have shown that the contraceptive drugs, BRCA1–2 mutations, and multiple ovulations can be associated with ovarian cancer [10–12]. Most of the ovarian cancers are sporadic, which are developed by the accumulation of genetic aberrations [13]. The serous borderline tumors and low-grade serous adenocarcinoma are mostly characterized by the BRAF and K-RAS mutations [14]. However, there are various molecular patterns associated with the heterogeneous biology of ovarian cancer in the case of histopathology and malignancy potential [15]. Like the foci of aggressive high-grade serous ovarian carcinoma (HGSOC), the STIC lesions were proliferative, as measured by Ki67 and p53 immunohistochemistry (IHC). DNA sequencing also showed that the majority of STIC clonal lesions harbor the same TP53 mutation as the simultaneous HGSOC [16, 17]. HGSOC is mainly characterized by mutations in TP53, mutations in the homologous recombination DNA repair pathway, and an extensive range of copy number changes. One of the most communal copy number changes in ovarian cancer is amplification at the 19q12 locus [18].

## Main text

### Staging and prognosis

The prognosis of ovarian cancer is directly related to the stage of tumor and tumor cells remaining after resection. Exploratory laparotomy paves the way for tumor staging and debulking [19]. Having command on the spread pattern of ovarian cancer is highly required in appropriate radiological determining, diagnosis, and surgery [20]. Computed tomography (CT) is used for ovarian cancer staging before the surgery and also for the determination of tumor relapse [21]. MRI and multi detector CT are efficient methods for ovarian cancer staging [19]. New methods such as the proteomics patterns and bioinformatic tools are also used to detect ovarian cancer in the early stages [22]. It has been observed that the PET/CT method can detect restaging modality of the ovarian tumor with a higher efficiency compared to the CT method [23]. It has been shown that the WB-DWI/MRI method has more prognostic accuracy in primary, peritoneal, and distant ovarian tumors compared with CT and FDG-PET/CT methods [23].

Due to lack of efficient early detection methods in ovarian cancer, around 70% of cases are diagnosed in advanced stages with poor prognosis [24]. Surgical resection along with platinum-based chemotherapy is a standard treatment option for ovarian cancer. According to ovarian cancer surgery guidelines (ESGO 2017), the objective of early surgery is complete resection of the macroscopic tumors [24]. After surgery, patients will be undergoing the platinum/taxane treatment as the first-line chemotherapeutic modality [25]. However, early surgery is not possible for the patients with advanced stages of tumor (III, IV), since their other organs such as the intestine and liver are involved. In such cases, a neoadjuvant chemotherapy will be done before the interval debulking surgery (IDS) [25]. The bevacizumab and paclitaxel are also the first line treatment options. Despite the successful results of early treatment, the majority of patients may have tumor recurrence [26]. Second-line chemotherapy is the principle approach in treatment of recurrent ovarian cancer. Combinational treatment of platinum and other drugs will be used for patients who have partially or highly sensitive tumors with recurrence after 6–12 months or more than 12 months [27]. The angiogenesis inhibitors can also be used beside these treatment methods in ovarian cancer patients. Since, Vascular Endothelial Growth Factor (VEGF) is a key factor during vascular progression inside the tumor, its inhibition can be resulted in tumor elimination [28]. Poly (ADP-Ribose) polymerase (PARP) inhibitors have been also used in advanced ovarian cancer patients with BRCA2 and BRCA1 mutations [29].

### Strategies for isolating and enriching CSCs

Cancer stem cells (CSCs) can be identified and isolated through different methods. Magnetic-activated cell storing method (MACS) and fluorescent-activated cell storing method (FACS) are efficient methods for isolation of CSCs from solid tumors based on cell surface or intra cellular markers [30]. MACS is a fast and easy method, but makes the separation in monoparameter form, whereas FACS is an expensive multiparameter isolation method [31]. Separation methods based on cell surface markers are commonly used for isolation of CSCs from the heterogeneous tumor cells. CD133 is one of the most common cell surface markers which is used for isolation of CSCs from various types of tumor cells such as breast cancer, glioblastoma, prostate cancer, colon cancer, and liver cancer [32]. It has been reported that the CD133 positive glioma stem cells (GSCs) were tumorigenic. The CD133+ and CD133- human lung cancer and mouse glioma cell lines were also tumorigenic with self-renewal and colonization abilities [33, 34]. Another study reported that the CD105 positive cells had higher CSC characteristics compared with CD105 positive cells following isolation using the MACS method [35]. CXCR4-positive cells sorted by FACS method had also higher ability in sphere formation and tumorigenesis in comparison with CXCR4-negative cells [36]. It has been shown that the CD133+/CD24+/CTR2+ cells had stem cell-like properties in renal cell carcinoma [37]. Several markers such as CD133, ALDH1/2, LY6A, LGR5, EpCAM, CD133, CD44, CD34, CD24, CD117, MyD88, and CDH1 were used for isolation of CSCs from the ovarian cell lines (Table 1) [38–47]. Another method of separating CSCs is based on Adlehyde dehydrogenase (ALDH) using Aldefluor method. This method allows single-cell imaging in monolayer cultures which can be a useful method in some cases. This method has higher stability and lower specificity compared with the cell surface methods [48]. Isolation based on ALDEFLUOR method from 6 ovarian cell lines and 8 ovarian cancer patients resulted in cells with higher sphere-formation ability, tumorigenicity, and invasiveness [49]. It has been also observed that the ALDH+CD133+ cells had a higher ability to create larger and faster tumors in xenograft mouse, and also create three dimensional sphere more efficiently compared with their negative counterparts in ovarian tumors [50]. Another method for separating CSCs is based on cell side population (SP) with the expression of ABC transporters using Hoechst 33342 dye-staining. In this method, SP cells exclude the Hoechst 33342 dye through a transporter. This is the mechanism to expel the chemotherapeutic drugs and creates resistance against chemotherapy [51]. This method is also used to isolate CSCs without a cell surface marker, however, it has lower specificity and purity and toxic effects on isolated cells compared with other methods. It was observed that the separated SP cells had high expression levels of CSC markers, ATP-binding cassette, ABCG2, nestin, and CD44 on SK-OV-3 ovarian cell line. These cells had a high self-renewal and proliferation ability [52].

**Table 1:** Surface markers used to isolate ovarian cancer stem cells

Surface marker	The distinctive feature of these cells	References
CD133^+^	Higher clonogenic and proliferative potentials recapitulate the tumor characteristics in NOD/SCID mice	[33, 34]
CD44^+^	Targeting CD44 by siRNA induced cell death and decreased the tumor	[35]
CD44^+^/CD117^+^	Recapitulate the original tumor in vivo	[33, 36]
CD44^+^/MyD88^+^	Presented stem-like characteristics, including constitutive NF-κB activity, high capacity for tumor reconstitution, resistance to chemotherapeutics ability to recapitulate the tumor in vivo and	[37]
CD44^+^/E-cadherin^−^/CD34^−^	Participate in neovascularization shorter tumor-free period in vivo and increased	[38]
CD44^+^/CD24^+^/EpCAM^+^ CD44^+^/CD24^−^	migration and invasion characteristics in vitro differentiation potential and drug resistance accompanied by higher invasion ability	[39] [40]

### Cancer stem cells and chemo resistance

There are many different biological aspects of the clinical development of ovarian cancer that support the role of cancer stem cells during tumor progression and disease survival. Ovarian cancer is often associated with peritoneal ascites, in which spheroids reside in tumor cells and survive and proliferate even in a non-adherent status. The anoikis resistance is a key feature of these stem cells [53]. Different aspects of stem cell biology, including quiescence, differentiation, EMT, and plasticity are regulated by different cellular niche components including non-stem cells, host cells, extracellular matrix, and soluble factors [54]. CSCs are a sub population of tumor cells with self-renewal properties which preserve the growth and heterogeneity of tumor during tumor relapse [55, 56]. During primary chemotherapeutic treatment, drug resistance in CSCs leads to the tumor relapse [57]. There are various percentages of CSCs in different tumor tissues. Ovarian CSCs were firstly observed through a multilayer spheroid culture. These spheroids have the ability to form new tumors in mice [58]. These cells have been suggested as key tumor-initiating factors which have an important role in tumor recurrence after chemotherapy through several chemotherapeutic resistance mechanisms. It has been shown that the CSCs have specific metabolic features such as higher glycolytic functions in comparison with differentiated tumor cells [59–62]. Such specific metabolic behaviors can be resulted in drug resistance. The rodent ovarian CSCs have higher glycolysis compared with parental cells, which can be associated with chemo resistance [63]. The CD44 + CD117 + ovarian CSCs also showed high levels of mitochondrial ROS, which suggested that the mitochondrial electron respiratory chain is mainly used to preserve the cells during nutrient starvation and stress conditions [64]. There are various drug resistance mechanisms in CSCs such as ABC transporters, Aldehyde dehydrogenase, DNA repair, and signaling pathways [65]. Hoechst 3342 is a method for CSCs detection, which is associated with the function of ABC transporters. P-glycoprotein (MDR1) and breast cancer resistance protein (ABCG2) are involved in dye exclusion and chemo resistance [66–68]. Although, Doxorubicin is excluded by both ABCB1 and ABCG2 [69], Paclitaxel is only pumped out by MDR1 [66, 69]. Therefore, higher expression of these transporters can be observed in different CSCs. The high levels of ABCG2 and ABCB1 have been observed in breast and ovarian CSCs, respectively [70, 71]. Moreover, it has been reported that there were high levels of ABCA1, ABCB5, and ABCC3/MRP3 expressions in ovarian tumor tissues [72] and high levels of ABCA1, ABCB1/MDR1/P-GP, and ABCG2/BCRP expression in ovarian CSCs [71, 73–75]. The correlation between ABC transporter type and chemo resistance mechanism is a critical issue to select a specific suppressor [76]. Aldehyde dehydrogenase (ALDH) is the other important mechanism of drug resistance among CSCs. There are different isoforms of human ALDH which are mainly expressed in kidney and liver [77]. ALDH over activity is considered as a prognostic marker for various cancers such as lung [78], breast [79], pancreas [80], intestine [81], and ovarian cancer [49]. The cyclophosphamide-resistance role of ALDH was identified in cyclophosphamide-resistant L1210 leukemic cell line which was retrievable by Disulfiram as an ALDH inhibitor [82]. The ALDH-mediated cyclophosphamide-resistance has been also reported in medullaoblastoma [83]. Moreover, ALDH is associated with CSCs phenotype, colony formation, self-renewal marker expression, tumor formation, and EMT process in ovarian cancer [84]. Therefore, ALDH inhibition may play an important role to sensitize the CSCs toward drugs. It has been reported that the ESA + CD44+ colon CSCs with high expression of ALDH were sensitized toward cyclophosphamide via ALDH1A1- siRNA [85]. The third mechanism which leads to chemo-resistance in CSCs is the role of B-cell lymphoma-2 (BCL-2) protein family. This protein family plays a significant role in balancing between survival, apoptosis, embryogenesis, neurogenesis, and hematopoiesis [86]. These proteins inhibit the BAX and BAK pro-apoptotic proteins to release Cytochrome c [87]. As a potential oncogene, BCL-2 protein is expressed in various neoplastic and hematopoietic lineage cells [88, 89]. The roles of BCL-2 family members have been widely studied in tumor cells survival and CSC biology. It has been reported that there were high levels of BCL-XL and BCL-2 expressions in quiescent leukemic CD34+ cells [90] and CD44+/CD24−/low breast CSCs [91]. Therefore, high levels of BCL-2 protein expression through signaling pathways are required for the CSCs survival and chemo-resistance. Decreased expression of BCL-2 is accompanied by increased sensitivity to FU-5 and Oxaliplatin [65]. Bcl-xl over expression has been observed in most of recurrent chemo-resistant ovarian cancers which were correlated with a shorter disease-free interval [92, 93]. Preclinical studies showed that the Bcl-xL inhibition increased chemo-sensitivity of ovarian cancer cells, which highlighted the inhibition of anti-apoptotic proteins as a promising therapeutic method for recurrent ovarian cancer [93, 94]. Signaling pathways such as WNT/β-catenin and NOTCH are also the other chemo-resistance processes in CSCs [95–99]. It has been observed that there was a correlation between WNT pathway and Cisplatin resistance OV6 + hepatic CSCs [100]. NOTCH signaling pathway has key functions in tumor progression, angiogenesis, epithelial-mesenchymal transition (EMT), and self-renewal [101–104]. It has been shown that the Notch 1 receptor knockdown or using γ-secretase inhibitors resulted in Oxaliplatin sensitization in intestine cancer cells [105]. Increased expression of Notch3 also plays a significant role in the biology of CSCs and Platinum resistance. The γ-secretase inhibitor (GSI) eliminates the CSCs via increasing the Platinum sensitivity. Altogether, the combination treatments including tumor resection and CSCs targeted therapy can be much more effective than routine treatments [106].

### Targeted therapy of ovarian cancer stem cells

Despite recent findings, ovarian cancer has still a high ratio of mortality. Regarding the importance of ovarian CSCs in drug resistance and tumor relapse, their elimination could be considered as an efficient treatment method to decrease chemo-resistance and relapse in ovarian cancer [107]. Therefore, three different strategies are applicable; signaling pathways could be a good choice, surface markers could be used as precise target and finally we will briefly discuss about some other methods to eradicate the CSC.

### Signaling pathways and targeted therapy

Targeting the signaling pathways is one of the best therapeutic options in CSCs. There are several key signaling pathways such as WNT, SONIC Hedgehog (SHH), NOTCH, PI3K/PTEN, and NF-kB which are associated with stem cell properties. Therefore, deregulation of these signaling pathways can be associated with CSCs survival [108]. We have summarized some recent studies in Table 2.

**Table 2:** Signaling pathways and targeted therapy substance

Targeted Pathway/s	Substance	Cancer/s type	Result/s	Clinical state
WNT	PRI-724	colon cancer	apoptosis induction	Experimental
WNT	LGK974	breast cancer, melanoma, pancreas cancer	determine the maximum tolerated dose and/or recommended dose for expansion, characterize the safety and tolerability, and assess preliminary antitumor activity	Phase 1
WNT	Ipafricept	Pancreas, ovarian cancers	determination of dose-limiting toxicities (DLTs)	Phase 1a/1b
SHH	Cyclopamine	ovarian cell lines, EX2, TOV112D, OV90, SKOV3	decreased spheroid formation	Experimental
SHH	Vismodegib	Basal tumor	Prevent metastatic cells	phase 1
SHH	Sonidegib	Basal Cell Carcinoma	Prevent metastasis	FDA Approved
SHH, PTCH	5E1 antibody	motor neuron	SMO inhibitors	Experimental
SHH	GDC-0449	ovarian cancer	SMO inhibitors	phase 2
NOTCH	Ƴ-secretase inhibitor, Cisplatin	ovarian cancer	increased chemo-sensitivity and decreased CSCs numbers	Experimental
NOTCH	Anti Jagged1	Taxane-resistant cell line	Docetaxel sensitivity and decreased tumor weight	Experimental
NOTCH	cediranib maleate	breast cancer, malignant melanoma, colorectal cancer, pancreatic cancer, kidney cancer, high grade glioma, non-small-cell lung cancer, and ovarian cancer	determine the tolerability, maximum tolerated dose and safety profile of RO4929097	phase 1
NOTCH	Ƴ-secretase inhibitor RO4929097	metastatic melanoma	Increased progression-free survival and 1-year overall survival rate	phase 1
NOTCH	Ƴ-secretase inhibitor of LY900009	ovarian cancer	inhibited plasma levels of amyloid-β peptide and inhibition of progression	phase 1
NOTCH	monoclonal antibodies against DLL4	ovarian tumors	increased apoptosis in tumor cells and reduced tumor weights	Experimental
NOTCH	Enoticumab	ovarian tumors	determine the safety, dose-limiting toxicities (DLT), pharmacokinetics (PK), and recommended phase II dose (RP2D) of enoticumab	Experimental
NOTCH	Demcizumab	ovarian tumors	increased apoptosis in tumor cells	Experimental
MAPK	Salinomycin	Ovarian cancer	decreased chemo-resistance	Experimental
MAPK	Salinomycin	OVCAR-3	decrease the CSCs	Experimental
EpCAM	Catumaxomab	ovarian malignant ascites patients	Decreases malignancy	phase III

### WNT signaling pathway

**Fig. 1:**
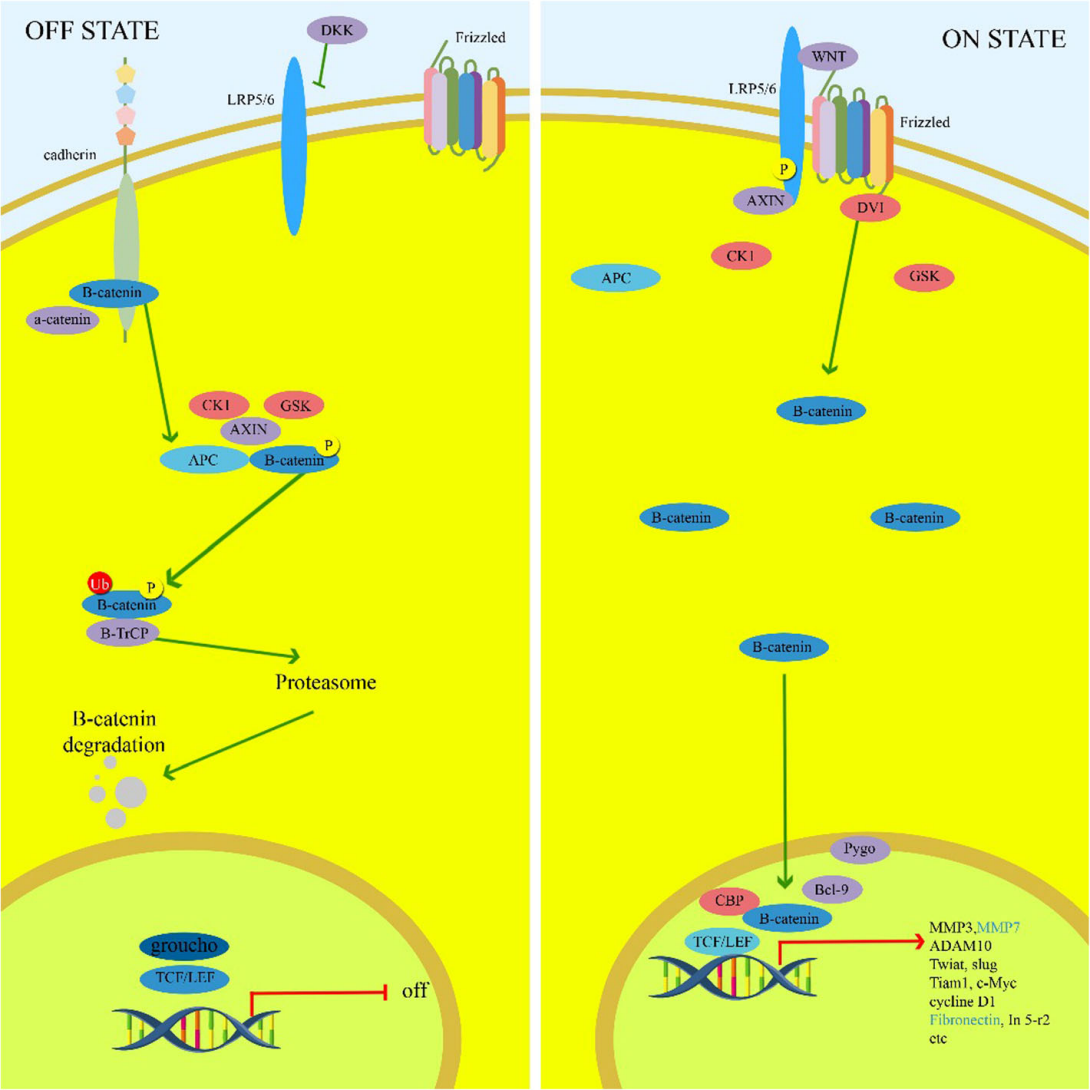
Schematic overview of the WNT signaling pathway. Wnt binds to (triggers) the receptor. Axin is removed from the “destruction complex.” β-catenin transfers into the nucleus, binds to a transcription factor on DNA, and stimulates transcription of a protein. Binding of Wnt to the receptors Frizzled (Fz) and LRP6 primes to inhibition of β-catenin degradation. β-catenin in turn interrelates with members of the TCF/Lef-1 family of transcription factors to co-activate target gene transcription

The canonical WNT signaling pathway is considered to be an important and protected pathway during embryogenesis and tissue homeostasis (Fig. 1) [109]. Deregulation of WNT pathway disrupts the natural growth and differentiation of colonic crypt stem cells, and increases the expression of target genes such as c-myc and cyclin D which results in a CSC phenotype [110]. Moreover, It has been observed that there was a significant correlation between WNT pathway and CSC properties in CD44+/CD133+ colon CSCs [111]. This pathway is also associated with chemoresistance in ovarian cancer [112]. WNT pathway plays an important role in the maintenance of stem cells in ovarian epithelium, while R-spondins activate this pathway through LGR receptors. The presence of LGR5 and LGR6 in regulation of epithelial stem cells and the chemoresistance of these cells plays an important role in ovarian cancer [113]. Elimination of CSCs by inhibition of WNT signaling can be considered as an efficient approach in tumor treatment [114]. PRI-724 inhibits the WNT pathway through CREB-binding protein which results in apoptosis induction in colon cancer cells [115]. LGK974 is a WNT inhibitor which is in phase 1 of the clinical trial and functioning in breast cancer, melanoma, and pancreatic cancer (NCT Number: NCT01351103). Ipafricept (OMP-54F28) as an Fc-Frizzled 8 receptor is also on 1a/1b phase pancreatic and ovarian cancers (NCT02092363, NCT02050178).

### Sonic hedgehog signaling pathway

**Fig. 2:**
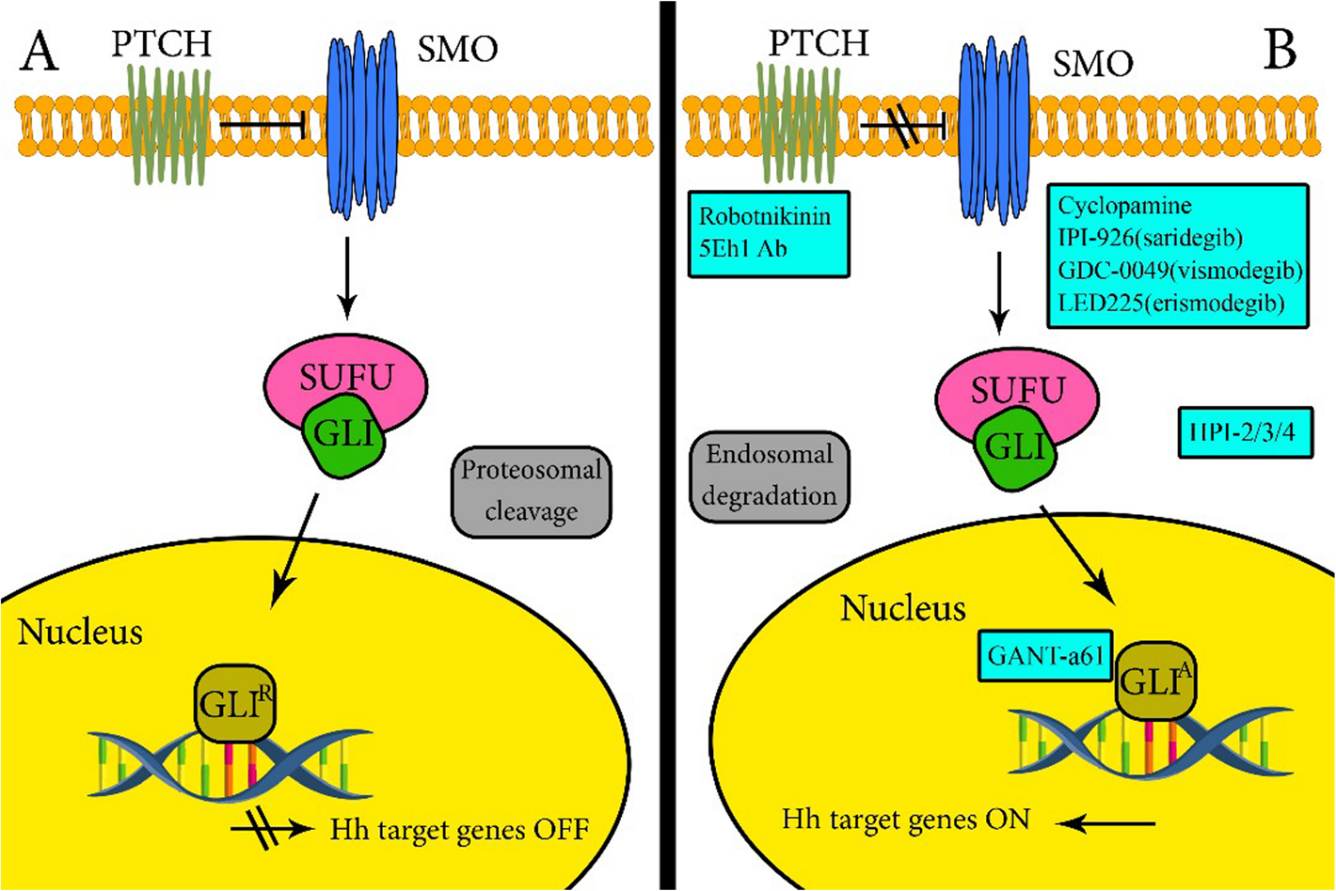
Schematic overview of the hedgehog signaling pathway and some inhibitors of the pathway in preclinical and clinical revisions. **a** In the absence of HH ligands, PTCH inhibits the role of SMO, and GLI proteins are changed by proteosomes to the transcriptional repressor form (GLIR). **b** Interaction of HH ligands with PTCH unrepresses SMO and creates activated GLI factors (GLIA) which encourage transcription of downstream HH genes. The bound of HH/PTHC complex develops adopted in the endosome and degraded

The SHH pathway has a critical role in a wide range of molecular and cellular processes such as embryogenesis, development, and adult tissue homeostasis (Fig. 2) [111, 116]. Deregulation of the SHH pathway has been reported in CSCs maintenance in several cancers such as breast cancer, pancreatic cancer, myeloma, lung cancer, glioblastoma, and CML [117–122]. SMO and Gli1 over expressions have been observed in myeloma CSCs [123]. Since the SHH pathway plays an important role in self-implantation of CSC and other characteristics of these cells, its inhibition may disrupt CSCs stemness through differentiation of these cells [124, 125]. Cyclopamine as a Hedgehog inhibitor is reported to decrease spheroid-formation (up to 10-folds) in several ovarian cell lines, such as EX2, TOV112D, OV90, and SKOV3 [126]. Vismodegib is a SHH inhibitor in phase 1 of the clinical trial, which targets SMO and is used against metastatic basal tumor cells [124, 125]. Sonidegib is also another SMO inhibitor approved by FDA for advanced BCC patients [127]. The 5E1 antibody inhibits conjunction of all three ligands of HH and PTCH [128, 129]. The GDC-0449 (Vismodegib derivative) and Sonidegic (LDE225) are also SMO inhibitors in phase 2 of the clinical trial (NCT00739661) and (NCT02195973) respectively in ovarian cancer.

### Notch signaling pathway

**Fig. 3:**
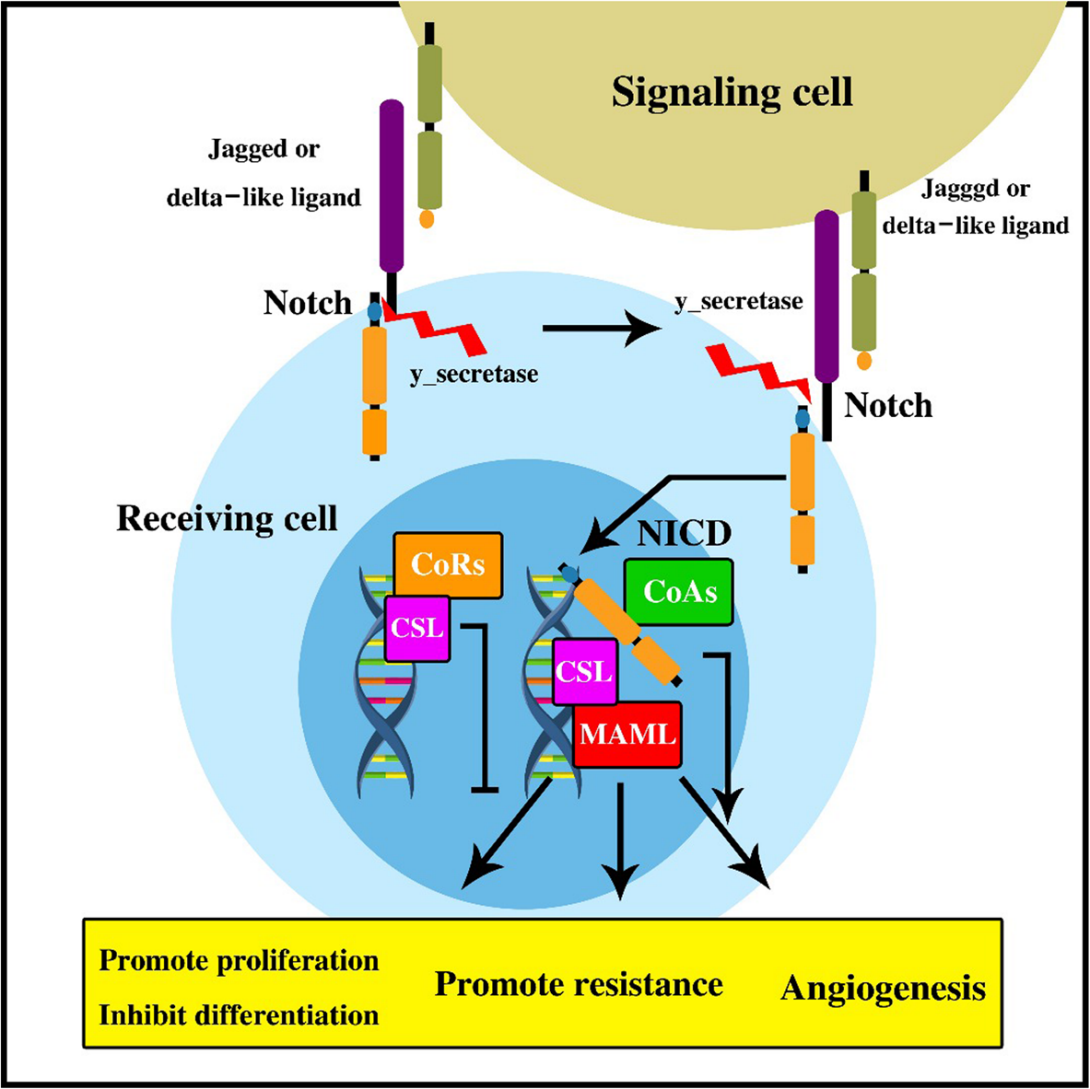
Schematic overview of the Notch signaling pathway. Ligands of the Jagged and Delta-like families interrelate with Notch family receptors on an adjacent cell. The Notch receptor exists at the cell surface as a proteolytically cleaved heterodimer containing of a large ectodomain and a membrane-tethered intracellular domain. The receptor-ligand interaction makes two additional proteolytic cleavages that free the Notch intracellular domain (NICD) from the cell membrane. The NICD moves to the nucleus, where it procedures a complex with the RBPJ protein, dislocating a histone deacetylase (HDAc)-co-repressor (CoR) complex from the RBPJ protein. Components of an activation complex, such as MAML1 and histone acetyltransferases (HAc), are engaged to the NICD-RBPJ complex, leading to the transcriptional activation of Notch target genes

Canonical NOTCH signaling pathway is one of the most important evolutionarily conserved pathways during development and adult tissue homeostasis (Fig. 3) [130, 131]. Deregulation of NOTCH signaling has an important role in the maintenance and survival of the CSCs in breast cancer, pancreatic cancer, and glioblastoma. Fascin is an Actin binding protein that is involved in the regulation of breast CSCs through NOTCH signaling pathway [132]. Therefore, Fascin knockdown decreases the expression of pluripotent genes and sphere formation in breast stem cell-like cells [132]. It has been reported that there were increased expression of NOTCH signaling components such as NOTCH 1, NOTCH3, JAG1, JAG2, and HES1 in pancreatic CSCs and Ƴ-secretase inhibitor decreased the CSC population and tumorsphere formation [133]. Activation of Notch signaling pathway by Delta/Serrate/Lag-2 peptide also is increased in the pancreatic CSCs tumorsphere, whereas NOTCH inhibition through HES1 knockdown decreased tumorsphere formation of pancreatic CSCs [133]. A combination of Ƴ-secretase inhibitor (GSI) and Cisplatin to target the NOTCH signaling pathway increased chemo-sensitivity and decreased CSCs numbers [106]. Another group targeted Jagged1 in the Taxane-resistant cells that caused Docetaxel sensitivity and decreased tumor weight [134]. A phase 1 clinical trial was done about a combination of Ƴ-secretase inhibitor RO4929097 and cediranib maleate (NCT01131234). The Ƴ-secretase inhibitor of LY900009 was also used in a phase 1 clinical trial for advanced ovarian cancer patients [135]. The other NOTCH inhibition method is using monoclonal antibodies against DLL4 (Delta-like lignad4) which prevents the ligand binding. Enoticumab (REGN421) is an anti-DLL4 antibody which is used in DLL4 over expressed ovarian tumors. Moreover, Demcizumab as an anti-DLL4 antibody has been also used in advanced ovarian tumors [136].

### Other signaling pathways

Salinomycin is an ionophore antibiotic which suppresses the ovarian CSCs through different mechanisms such as ABC transporters and MAPK pathway which leads to a decreased chemo-resistance [137, 138]. Also these antibiotics decrease the CSCs numbers in OVCAR-3 cell line through down regulation of Bcl-2 [139]. Catumaxomab is also a monoclonal antibody used for epithelial cell adhesion molecule (EpCAM), which is in the clinical trial phase III among ovarian malignant ascites patients [140, 141].

### Eliminating CSCs by targeting their surface markers

Surface markers of the CSCs, like CD24, CD44, CD117 and CD133 can be targeted by several strategies [142]. The CD44+ SKOV3 cell lines were targeted by hyaluronic acid-paclitaxel (HA-TXL) which resulted in decreased tumor weight and nodules [143]. In another report, CD133+ OVCAR5-luc cells were targeted that resulted in a considerable decrease in tumor progression [144]. The CD24 inhibition decreased cell viability through apoptosis induction in SKOV3 cell line, and restricted the tumor growth in nude mice [145]. There is a correlation between CD117 surface marker and drug resistance in ovarian cancer [146]. Activation of Wnt/β –catenin-ABCG2 pathway for Cisplatin/Paclitaxel resistance is occurred by CD117 in ovarian CSC. The Imatinib Mesylate as a CD117 inhibitor has been used to treat various tumor types and chemo-resistant ovarian tumors [147, 148]. The growth of CD44+ and CD117+ chemo resistant ovarian CSCs were also inhibited by Paclitaxel and Salinomycin treatments [149]. Metformin is another drug associated with increased 5-year survival rate of ovarian cancer patients. It has been observed that the Metformin inhibited CD44+ and CD117+ CSCs and EMT process in SKOV3 and A2780 cell lines [150]. Another group showed that the Metformin decreased ALDH+ CSC population and angiogenesis [151]. *Clostridium perfringens* Enteroxin (CPE) can also be used to eliminate the chemo-resistant CD44+ ovarian CSCs in Xenograft mouse model [152].

### Other potential strategies to eliminate CSCs (differentiation therapy, niches and miRNAs)

Differentiation therapy is one other method to eradicate the CSC [153]. Retinoic acids are the only factors that have been used in clinical trials of differentiation therapy [154]. It has been shown that the Carboplatin in combination with Novel Retinoid Compounds 3 efficiently reduced the growth of ovarian CSCs [155].

The tumorigenic ability of ovarian tumor cells is associated with niches derived from human embryonic stem cells [156]. Hypoxic Niches also provide suitable conditions to obtain the properties of ovarian cancerous stemness [157]. Therefore, these Niches can be considered as appropriate treatment targets.

MiRNAs are a group of noncoding RNAs, which are involved in tumor progression [158]. There are different miRNA expression profiles between normal and cancer stem cells [159, 160]. It has been reported that there was increased levels of miR-214 expression in ovarian CSCs which was correlated with self-renewal and chemo resistance [161]. MiR-199a also prevents the tumor growth and increases the sensitivity toward Cisplatin, Paclitaxel, and Adriamycin through down regulation of CD44 in ovarian CSCs [162]. It has been also shown that the miR-200a decreased the migration of ovarian CD133 + CSCs [163].

## Conclusions

Regarding the importance of CSCs in ovarian cancer progression and metastasis, it is required to clarify the molecular biology of CSCs to introduce novel markers for the elimination of such cells in ovarian tumors. Indeed, molecular targeted therapy against the CSCs will improve patient’s survival and decrease the tumor relapse among ovarian cancer patients. According to the recent studies, it was concluded that a combination therapy including tumor resection and CSC targeted therapy can be one of the most efficient anti-cancer therapeutic methods against ovarian tumors.

## Data Availability

The datasets used and/or analyzed during the current study are available from the corresponding author on reasonable request.

## Abbreviations

ABCG2: ATP-binding cassette sub-family G member 2
ALDH: Adlehyde dehydrogenase
BCL-2: B-cell lymphoma-2
CPE: *Clostridium perfringens* Enteroxin
CSCs: Cancer stem cells
CT: Computed tomography
FACS: Fluorescent-activated cell storing method
GSCs: glioma stem cells
GSI: γ-secretase inhibitor
IDS: Interval debulking surgery
MACS: Magnetic-activated cell storing method
OSE: Surface epithelial cells
SP: Side population
STIC: Intra epithelial carcinomas
VEGF: Vascular Endothelial Growth Factor
